# Awareness, treatment and control of hypertension among hypertensive patients aged 18 to 59 years old in the northeast of China

**DOI:** 10.1038/s41598-018-34923-5

**Published:** 2018-11-19

**Authors:** Xin Lv, Huikun Niu, Yangming Qu, Meiqi Li, Lu Li, Xiaoyu Ma, Shan Jiang, Chunshi Gao, Rui Wang, Peng Zhang, Bo Li

**Affiliations:** 1 Department of Epidemiology and Biostatistics, School of Public Health, Jilin University, Changchun, 130021 China; 2 Department of Thoracic Surgery, the First Hospital of Jilin University, Changchun, 130021 China; 3 Department of Neurology, Stroke Center, the First Hospital of Jilin University, Changchun, 130021 China; 4 Jilin Women and Children Health Hospital, Changchun, 130061 China

## Abstract

Hypertension not only has a high prevalence, but also brings disease burden to the affected patients. To assess the level of awareness, treatment and control of hypertension in the northeast of China, we investigated the rates of awareness, treatment and control of hypertension and identified its related factors among hypertensive patients aged 18 to 59 years old in Jilin, China. The data (n = 4632) for the present study were extracted from a cross-sectional study in Jilin. Among individuals with hypertension, the standardized rates of awareness, treatment and control of hypertension were 44.9%, 36.5%, 24.3%, respectively. The rates of awareness and treatment of hypertension among middle aged patients were higher than those among young patients. Compared to patients with normal Body Mass index (BMI), obese hypertensive patients had a higher rate of treatment (43.7% vs. 25.9%) and a lower rate of control (18.9% vs. 29.6%). Compared to patients with normal BMI, patients who were obese were more likely to take measures to treat hypertension (OR = 2.50, 95%CI: 2.05–3.05); but were less likely to have well-controlled blood pressure (OR = 0.55, 95%CI: 0.40–0.78). BMI is one of the influencing factors of hypertension awareness, treatment and control among patients 18 to 59 years old with hypertension.

## Introduction

Cardiovascular diseases (CVDs), a subtype of major non-communicable diseases, account for approximately one third of total global deaths^1,2^. It is predicted that CVD-related global deaths will climb to more than 23.3 million by 2030^3^. Hypertension is a key risk factor for cardiovascular disease^1^. In 2008, approximately 40% of adults over 25 years of age had hypertension^4^. The prevalence of hypertension will be likely to increase 29% by 2025^5^. Worldwide, there are 9.4 million hypertension-related deaths^6^. Hypertension is the cause of at least 45% of ischemic heart disease deaths, and 51% of stroke deaths^7^. In China, the prevalence of hypertension increased between 1959^8^ and 2002^9^ according to the National Nutrition and Health Survey (NNHS). It is estimated that 200 million people in China suffer from hypertension^10^. The prevalence of hypertension in north of China (including the northeast, middle north and northwest) is higher than that in south of China (including the east, middle south and southwest)^11^. The prevalence of hypertension in the Jilin province, which located in the northeast of China, was 37.3% in a reported study conducted in 2012^12^.

Hypertension is one of the leading contributors to the global disease burden (7.0% of global disability-adjusted-life-years)^6^. It is estimated that 10% of healthcare spending is directly related to increased blood pressure and its complications^13^. Hypertension decreases the quality of life and increases the financial burden on health care systems^4^. Detecting hypertension early and taking measures to control it may be a cost-effective way to reduce the hypertension-related disease burden^1^. In China, the status of the awareness, treatment and control of hypertension has increased with time. From 1991 to 2011, the rate of awareness of hypertension increased from 33.7 to 54.9%, the rate of treatment increased from 19.9 to 45.9%, and the rate of control increased from 12.6 to 30.1% among hypertension patients^14^. However, the rates of awareness, treatment and control of hypertension are still lower than that of other countries^15–18^.

Mean systolic blood pressure decreased in older adults, but increased in younger adults, during the 24 years from 1989 to 2013^19^. Hypertension is increasingly affecting people younger than 50 years old, many of whom die prematurely^20^. The rates of awareness, treatment and control of hypertension are at low levels among young and middle-aged hypertensive patients^14,21^. Early intervention programs have impressive influence on the hypertension awareness, treatment and control^22^. Accordingly, it is necessary to study the related factors of awareness, treatment and control among young and middle-aged hypertensive patients. Previous studies in Jilin province paid more attention to the prevalence and the risk factors of hypertension^23,24^, but epidemiological investigation of the awareness, treatment and control among hypertensive patients is limited, especially research on young and middle-aged patients. This study investigated the rates of awareness, treatment and control status among hypertensive patients aged 18 to 59 years old and identified the related factors for hypertension awareness, treatment and control.

## Results

### Characteristics of hypertensive patients

As shown in Table 1, a total of 4632(2556 males and 2076 females) participants completed the questionnaire and all examinations. To make the sampling survey more representatively, we used complex weighted computation throughout the data analysis. The median age of the participants was 49 years old (interquartile range, 43–54 years old). The percent of participants in the young and middle-aged groups were 41.7% and 58.3%, respectively. There were more male patients than female patients in this study (61.5% vs. 38.5%). The median BMI of participants was 25.55 kg/m^2^ (interquartile range, 23.39–28.07 kg/m^2^); 30.1% of the participants with normal BMI, 42.2% of the participants were overweight and 26.8% of the participants were obese.

**Table 1:** Characteristics of Hypertension patients (n = 4632)[M(Q_25_,Q_75_)/n(%)].

Category	Median(Interquartile range)/Frequency	Estimated population
Systolic blood pressure(mmHg)	146(140,158)	4059
Diastolic blood pressure(mmHg)	91(85,97)	4059
Age(years)	49(43,54)	4059
Age group		
Young	1418(41.7)	1694
Middle-aged	3214(58.3)	2365
Sex		
Female	2076(38.5)	1561
Male	2556(61.5)	2498
Region		
Rural	2333(45.9)	1862
Urban	2299(54.1)	2197
Education level		
Primary school or low(≤6 years)	1205(22.0)	894
Junior high school(7~9 years)	1390(31.2)	1267
Senior high school(10~12 years)	1416(31.3)	1270
University or above(>12 years)	621(15.5)	628
Marital status		
Married	4209(88.2)	3580
Single	179(6.9)	282
Divorced or separated	102(2.4)	96
widowed	142(2.5)	101
Occupation		
Manual workers	2807(60.4)	2448
Mental workers	915(21.4)	870
Retired	342(6.7)	274
Others	568(11.5)	467
Income(Yuan)		
<500	962(16.8)	685
500~	940(19.4)	785
1000~	1480(33.0)	1339
2000~	823(20.4)	830
3000~	427(10.4)	420
Body Mass index(kg/m^2^)	25.55(23.39,28.07)	4059
BMI group		
Normal	1411(30.1)	1223
Underweight	43(0.9)	38
Overweight	1984(42.2)	1713
Obesity	1194(26.8)	1085
Family history of circulation system diseases		
No	1955(41.9)	1699
Yes	2677(58.1)	2360
Smoking		
Never	2553(52.7)	2139
Current	1655(38.0)	1544
Past	424(9.3)	376
Consuming alcohol products		
No	2744(54.8)	2224
Yes	1888(45.2)	1835
Physical exercises		
Often	1340(28.2)	1143
Sometimes	1087(25.6)	1041
Never or rare	2205(46.2)	1875

### Awareness, treatment and control of hypertension

Table 2 shows the rates of awareness, treatment and control of hypertension of patients in different categories. The rates of awareness, treatment and control of hypertension were 44.9%, 36.5% and 24.3%, respectively. Middle-aged hypertensive patients had higher rates of hypertensive awareness and treatment than young patients (54.2% vs 31.9%, *P* < 0.001and 45.3% vs 24.3%, *P* < 0.001, respectively). The incidence of awareness and treatment was lower in men than in women (38.4% vs 55.3%, *P* < 0.001and 29.7% vs 47.5%, *P* < 0.001, respectively). Among the target population, different BMI groups had different levels of hypertension awareness, treatment and control status (*P* < 0.05). Obese patients had higher rates of hypertension awareness (52.6% vs. 33.0%) and treatment (43.7% vs. 25.9%) than patients with normal BMI.

**Table 2:** Comparison of awareness, treatment and control of hypertension between various factors.

Category	Subcategory	Awareness		Treatment		Control among treated	
		n (%)	Estimated population	n(%)	Estimated population	n(%)	Estimated population
total		2227(44.9)	1823	1832(36.5)	1483	420(24.3)	361
Age group	Young	469(31.9)	541	361(24.3)	412	92(27.5)	113
	Middle-aged	1758(54.2)	1282	1471(45.3)	1071	328(23.1)	248
*χ* ^2^		225.436		214.418		3.940	
*P*		<0.001		<0.001		0.152	
Sex	Female	1198(55.3)	863	1028(47.5)	742	255(26.4)	196
	Male	1029(38.4)	960	804(29.7)	741	165(22.2)	165
*χ* ^2^		125.890		150.278		4.287	
*P*		<0.001		<0.001		0.086	
Region	Rural	1211(48.0)	894	990(38.5)	718	219(24.0)	173
	Urban	1016(42.3)	929	842(34.8)	765	201(24.6)	188
*χ* ^2^		14.927		6.888		0.065	
*P*		0.001		0.019		0.834	
Education level	Primary school or low( ≤ 6 years)	668(52.9)	473	552(43.0)	385	125(25.7)	99
	Junior high school(7~9 years)	620(41.2)	522	511(33.9)	429	135(28.0)	120
	Senior high school(10~12 years)	667(45.8)	582	552(37.5)	476	119(22.4)	107
	University or above(>12 years)	272(39.2)	246	217(30.7)	193	41(17.9)	35
*χ* ^2^		44.633		33.886		10.853	
*P*		<0.001		<0.001		0.058	
Marital status	Married	2049(46.6)	1670	1684(38.0)	1360	387(24.6)	335
	Single	39(16.3)	46	32(12.4)	35	8(22.2)	8
	Divorced or separated	49(48.0)	46	41(38.3)	37	10(24.0)	9
	Widowed	90(60.1)	61	75(50.5)	51	15(17.8)	9
*χ* ^2^		122.740		94.207		1.634	
*P*		<0.001		<0.001		0.714	
Occupation	Manual workers	1276(41.7)	1021	1029(33.4)	817	235(24.2)	197
	Mental workers	420(43.8)	382	334(34.2)	298	67(20.3)	61
	Retired	206(60.2)	165	187(54.9)	151	49(28.5)	43
	Others	325(54.6)	255	282(46.5)	217	69(27.5)	60
*χ* ^2^		62.011		82.587		6.426	
*P*		<0.001		<0.001		0.194	
Income(Yuan)	<500	526(51.6)	354	441(42.8)	293	97(23.7)	70
	500~	484(50.1)	394	400(41.2)	324	86(25.1)	81
	1000~	677(42.7)	572	553(34.6)	463	138(24.7)	114
	2000~	331(37.3)	309	268(30.2)	250	61(25.8)	65
	3000~	209(46.2)	194	170(36.3)	153	38(20.3)	31
*χ* ^2^		49.737		40.645		2.269	
*P*		<0.001		<0.001		0.802	
Body Mass index(kg/m^2^)	Normal	518(33.0)	403	401(25.9)	317	109(29.6)	94
	Underweight	17(35.4)	14	13(27.0)	10	5(45.7)	5
	Overweight	1015(48.8)	836	842(39.8)	682	197(25.3)	172
	Obesity	677(52.6)	570	576(43.7)	474	109(18.9)	70
*χ* ^2^		123.352		105.690		18.791	
*P*		<0.001		<0.001		0.002	
Family history of circulation system diseases	No	757(35.1)	596	600(27.6)	470	147(26.0)	122
	Yes	1470(52.0)	1227	1232(42.9)	1013	273(23.5)	239
*χ* ^2^		129.788		113.778		1.287	
*P*		<0.001		<0.001		0.328	
Smoking	Never	1297(47.4)	1015	1090(39.5)	845	251(24.9)	210
	Current	700(39.8)	614	554(31.1)	480	127(23.8)	114
	Past	230(51.6)	194	188(42.1)	158	42(23.0)	37
*χ* ^2^		32.909		37.566		0.469	
*P*		<0.001		<0.001		0.841	
Consuming alcohol products	No	1523(52.5)	1168	1308(45.1)	1004	320(25.9)	260
	Yes	704(35.7)	655	524(26.1)	479	100(21.0)	101
*χ* ^2^		131.583		179.541		5.214	
*P*		<0.001		<0.001		0.059	
Physical exercises	Often	748 (52.6)	601	646(45.5)	520	155(25.0)	130
	Sometimes	469(39.4)	411	377(30.7)	319	88(23.9)	76
	Never or rare	1010(43.2)	811	809(34.3)	644	177(24.0)	155
*χ* ^2^		47.870		66.987		0.261	
*P*		<0.001		<0.001		0.909	

### Factors associated with the awareness, treatment and control of hypertension

Table 3 shows the related factors associated with the awareness of hypertension. Middle-aged hypertensive patients were more likely to be aware of hypertension than young patients (OR = 2.07, 95%CI: 1.76–2.44). Male patients were less likely to be aware of hypertension than female (OR = 0.66, 95%CI: 0.54–0.80). Urban patients were less likely to be aware of hypertension than rural patients (OR = 0.73, 95%CI: 0.63–0.84). Single hypertensive patients were less likely to be aware of their blood pressure condition (OR = 0.40, 95%CI: 0.25–0.62) compared to patients who were married. Additionally, overweight patients (OR = 1.89, 95%CI: 1.60–2.24) and obesity patients (OR = 2.58, 95%CI: 2.13–3.12) were more likely to be aware of hypertension than those with normal BMI. Subjects who consumed alcohol products (OR = 0.64, 95%CI: 0.53–0.76) were less likely to be aware of hypertension than those who did not.

**Table 3:** Factors associated with awareness of hypertension among hypertensive patients.

Category	Subcategory	Awareness		
		OR	95.0% CI	*P*
Age group	Young	1		
	Middle-aged	2.07	1.76–2.44	<0.001
Sex	Female	1		
	Male	0.66	0.54–0.80	<0.001
Region	Rural	1		
	Urban	0.73	0.63–0.84	<0.001
Marital status	Married	1		
	Single	0.40	0.25–0.62	<0.001
	Divorced or separated	1.23	0.79–2.04	0.333
	Widowed	1.33	0.88–2.21	0.184
Occupation	Manual workers	1		
	Mental workers	1.25	1.04–1.50	0.018
	Retired	1.23	0.93–1.64	0.144
	Others	1.41	1.11–1.79	0.004
Body Mass index(kg/m^2^)	Normal	1		
	Underweight	1.40	0.70–2.77	0.342
	Overweight	1.89	1.60–2.24	<0.001
	Obesity	2.58	2.13–3.12	<0.001
Family history of circulation system diseases	No	1		
	Yes	2.02	1.76–2.33	<0.001
Smoking	Never	1		
	Current	1.44	1.20–1.72	<0.001
	Past	1.61	1.25–2.08	<0.001
Consuming alcohol products	No	1		
	Yes	0.64	0.53–0.76	<0.001
Physical exercises	Often	1		
	Sometimes	0.75	0.62–0.91	0.004
	Never or rare	0.73	0.62–0.86	<0.001

*Adjusted for gender, age and region (rural or urban).

The results from the logistic regression model analyzing factors associated with hypertension treatment are shown in Table 4. The model showed that male patients(OR = 0.65,95%CI:0.54–0.79), urban patients(OR = 0.79,95%CI:0.68–0.92), single patients(OR = 0.43,95%CI:0.26–0.69) and individuals who consumed alcohol products(OR = 0.54,95%CI: 0.45–0.65) were less likely to take measures to treat hypertension. It was noted that retired patients (OR = 1.35, 95%CI:1.02–1.79) and patients who were overweight (OR = 1.84, 95%CI:1.55–2.20)or obese (OR = 2.50, 95%CI:2.05–3.05) were more likely to take measures to treat hypertension.

**Table 4:** Factors associated with treatment of hypertension among hypertensive patients.

Category	Subcategory	Treatment		
		OR	95.0% CI	*P*
Age group	Young	1		
	Middle-aged	2.03	1.71–2.41	<0.001
Sex	Female	1		
	Male	0.65	0.54–0.79	<0.001
Region	Rural	1		
	Urban	0.79	0.68–0.92	0.002
Marital status	Married	1		
	Single	0.43	0.26–0.69	0.001
	Divorced or separated	1.22	0.74–2.01	0.429
	Widowed	1.21	0.80–1.84	0.374
Occupation	Manual workers	1		
	Mental workers	1.18	0.98–1.43	0.084
	Retired	1.35	1.02–1.79	0.035
	Others	1.39	1.09–1.76	0.007
Body Mass index(kg/m^2^)	Normal	1		
	Underweight	1.28	0.62–2.65	0.498
	Overweight	1.84	1.55–2.20	<0.001
	Obesity	2.50	2.05–3.05	<0.001
Family history of circulation system diseases	No	1		
	Yes	1.97	1.70–2.27	<0.001
Smoking	Never	1		
	Current	1.45	1.20–1.75	<0.001
	Past	1.59	1.23–2.06	<0.001
Consuming alcohol products	No	1		
	Yes	0.54	0.45–0.65	<0.001
Physical exercises	Often	1		
	Sometimes	0.68	0.56–0.83	<0.001
	Never or rare	0.66	0.56–0.79	<0.001

*Adjusted for gender, age and region (rural or urban).

Table 5 shows that gender and urban-rural distribution were not influencing factors of blood pressure control among hypertensive patients. Middle-aged patients were less likely to have well-control BP (OR = 0.72, 95%CI: 0.54–0.95). Obesity (OR = 0.54, 95%CI: 0.39~0.76) was negatively associated with maintaining blood pressure in the appropriate range (BP <140/90 mmHg).

**Table 5:** Factors associated with control of hypertension among hypertensive patients.

Category	Subcategory	Control among treated		
		OR	95.0% CI	*P*
Age group	Young	1		
	Middle-aged	0.72	0.54–0.95	0.022
Sex	Female	1		
	Male	0.82	0.64–1.05	0.120
Region	Rural	1		
	Urban	1.19	0.90–1.56	0.220
Education level	Primary school or low(≤6 years)	1		
	Junior high school(7~9 years)	1.064	0.78–1.46	0.698
	Senior high school(10~12 years)	0.79	0.56–1.11	0.172
	University or above( > 12 years)	0.58	0.35–0.95	0.030
Body Mass index(kg/m^2^)	Normal	1		
	Underweight	1.73	0.64–4.72	0.283
	Overweight	0.82	0.61–1.10	0.180
	Obesity	0.54	0.39–0.76	<0.001

*Adjusted for gender, age and region (rural or urban).

## Discussion

In this survey, there were 4632 respondents. Among 18~59 years old respondents with hypertension, 44.9%were aware of their condition; approximately 36.5% of participants used antihypertensive medication while 24.3% had their BP controlled well.

Age is one of the factors that affect the awareness, treatment and control of hypertension in hypertensive patients. In our study, the level of hypertensive awareness and treatment were significantly lower in younger patients than in middle-aged patients, but there was no significant difference in the level of hypertension control. This result was consistent with a previous study in Kazakhstan^25^. It might be considered that young patients have more adverse life styles and habits than older patients. A study in Iran shows that the rates of awareness, treatment and control of hypertension increased with age^26^, and a study in Senegal has similar results^27^. Age is a powerful risk factor for the development of hypertension^28^. Management of age-associated hypertension may be a key issue in the prevention of cardiovascular events^29^. Gender differences have been shown in the management of hypertension^30^. We found that gender is associated with hypertension awareness and treatment; men had larger hypertension prevalence than women among young and middle-aged hypertensive patients but had a lower incidence of awareness and treatment of hypertension than women. This result is similar to those reported in a study in north China^31^, a study in Korea^32^ and a study in Malaysian^33^. There are some researchers in China who concluded that the rates of awareness, treatment and control of hypertension in women were higher than that in men^14,34,35^. However, Ursua, R. *et al*.^36^ found that the rates of awareness, treatment and control of hypertension were not significantly distinct between the sexes. The impact of sex on hypertension and antihypertensive therapy remains unclear. It has been estimated that the overall life expectancy for 50-year-old individuals is 4.9 years shorter for hypertensive women and 5.1 years shorter for hypertensive men compared to normotensive individuals of the same age^37^. Blood pressure management in male hypertensive patients should be strengthened. Location and marital status are associated with hypertension awareness and treatment. Previous studies have found that patients who lived in urban areas had more opportunities to be aware of their blood pressure level than those who lived in rural areas^18,38,39^. Interestingly, our study shows a completely opposite result. In our study, we found that patients who came from urban areas might be less likely to be aware of their blood pressure condition and take measures to treat hypertension than those came from rural areas. The reason for this phenomenon was that there were a larger proportion of rural subjects in our research. A previous study indicated that marital status was associated with hypertension awareness and control^40^. We found that single hypertensive patients were less likely to be aware of their blood pressure condition and be on treatment than patients who were married.

Different BMI groups are associated with hypertension awareness and treatment; obesity is negatively associated with hypertension control. From our research, we found that individuals who were overweight or obese were more likely to be aware of their conditions and be on treatment than those with normal BMI. Pereira, M. *et al*.^41^ also suggest that awareness of hypertension is directly associated with BMI in both males and females; participants who are overweight or obese are more likely to be aware of hypertension and take hypertension medication. Supiyev, A. *et al*.^25^ and Evelyn, P. *et al*.^42^ found that obese patients are more likely to be aware of their hypertension. Lei, W. *et al*.^43^ reported that a higher BMI was significantly associated with hypertension awareness and treatment in both 2001 and 2010 among an urban elderly population. Moreover, patients who were obese may not decrease their BP below 140/90 mmHg compared to those with normal BMI. Work by Pereira, M. *et al*.^41^ also suggested that BMI is associated with a lower likelihood of blood pressure control. Hu, M. *et al*.^44^ have a different view about the relationship between obesity and blood pressure control; they suggest that it is easier for obese patients to have well-control blood pressure. The prevalence of hypertension increases with increasing BMI^21^. The current study found that BMI was a more reliable predictor of hypertension for senior adults^45^. Obesity increases the risk of treatment-resistant arterial hypertension^46^. Thus, obese patients with hypertension should pay more attention to the control of their hypertension. Clinical trials have demonstrated that weight loss of approximately 10% of a patient’s original body weight, by calorie restriction and/or increased activity, is an effective means to achieve clinically meaningful reductions in blood pressure and mortality from cardiovascular disease^47^. Reductions in BMI are associated with decreases in carotid intima media thickness and improvements in the cardiovascular risk-factor profile, suggesting that weight loss is worthwhile because it might result in long-term benefit^48^.

Alcohol consumption is related to hypertension awareness, treatment and control. Individuals who consumed alcohol products could be more likely to ignore hypertension awareness and treatment than those who do not consumed alcohol. Yang, L. *et al*.^35^ concluded that abstaining from alcohol is associated with hypertension treatment and affects the control of hypertension. However, Hu, M. *et al*. have a different perspective and report that alcohol consumption is associated with a higher rate of hypertension awareness^44^.

The sample for this study was extracted from a complex sampling scheme, and the sample size was large. However, there are still some limitations to our study. This study was a cross-sectional study. The definitions of hypertension awareness and anti-hypertensive treatments were all acquired from self-reported questionnaires, which can cause reporting bias. The life styles (smoking, consuming alcohol products, physical exercises) were also self-reported and provided from memory, which may cause some recall bias.

In conclusion, the rates of awareness and treatment of hypertension among middle aged patients were higher than that in young patients. The condition of awareness and treatment among male hypertensive patients were poor. Obese hypertensive patients may be less likely to maintain blood pressure within the normal range. Young hypertensive patients, male hypertensive patients and obese hypertensive patients may benefit from improved hypertension management.

## Methods

### Population sample

**Figure 1:**
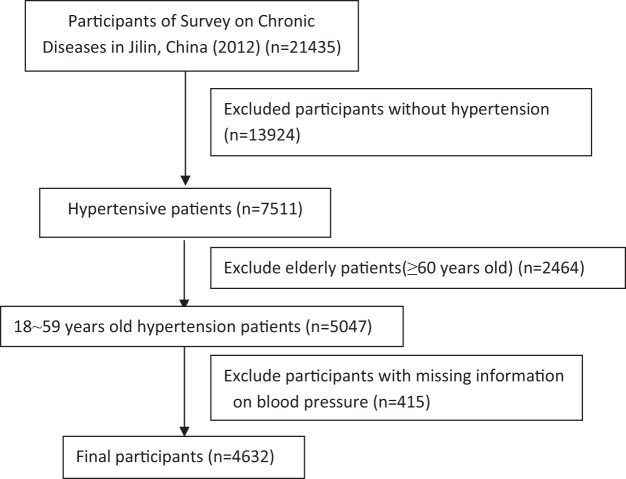
Flowchart of the participants included in or excluded from the present study.

This study is based on the 2012 Survey on Chronic Diseases in Jilin, China^49,50^. The survey data were collected from a multistage stratified cluster sample. The sample size has been estimated according to the prevalence of chronic disease in Jilin province. The sampling process included five stages. In the first stage, 9 cities (Changchun, Jilin, Siping, Liaoyuan,Tonghua, Baishan, Songyuan, Baicheng and Yanbian) were chosen from 32 districts/counties for inclusion in the study; they were identified by the proportion of the population, the location of the whole province and the race of their residents. In the second stage, towns were selected by stratified random sampling based on the size of the district. In the third stage, three committees which were near each selected towns were chosen by stratified random sampling. In the fourth stage, one village which was close to the selected committee was chosen by simple random sampling. In the fifth stage, individuals were chosen by cluster random sampling. Finally, 23050 subjects from 32 districts/counties were enrolled, and 21435 participants completed the survey (response rate: 84.9%)^49^. The survey included three aspects: a face-to-face questionnaire interview by trained investigators, a physical examination (measurement of height, weight and blood pressure) and a laboratory examination. The study was performed in accordance with the principles of the Declaration of Helsinki. Data in this study came from the epidemiologic investigation of the project (Survey on Chronic Diseases in Jilin, China, 2012), specifically from the part which contained information on participants with hypertension that were 18 to 59 years old. Ultimately, 4632 participants were included in the present study. The flowchart of the participants included in or excluded from the present study is shown in Fig. 1.

### Definition

Hypertension was defined as any blood pressure where the mean systolic blood pressure was equal to or higher than 140 mmHg and/or the mean diastolic blood pressure was higher than 90 mmHg, and/or the self-reported use of antihypertensive medication in the past two weeks after having had a diagnosis of hypertension by a physician at the county-level or above^51^. After at least 5 minutes rest first, blood pressure was measured two times by an electric sphygmomanometer (HEM-7200, OMRON, Dalian, China) on the arm in the sitting position; the final blood pressure was the average of the two measurement results.

Awareness of hypertension was defined as a self-reported diagnosis of hypertension by physicians. Individuals who had used anti-hypertension medications in the past 2 weeks prior to the interview were classified as being on treatment. Control was defined as systolic blood pressure lower than 140 mm Hg and diastolic blood pressure lower than 90 mm Hg among hypertensive patients who were being treated.

Subjects aged 18–44 years old were defined as the young group and subjects aged 45–59 years old were defined as the middle-aged group^50^. Education levels were divided into four groups: primary school or lower (including those who never attended school and those who only finished elementary school, years of education ≤6), junior high school (6 < years of education ≤9), senior high school (including those who attended senior high school and those who attended secondary vocational education, 9 < years of education ≤12) and university or above(including undergraduate and graduate levels of education, years of education >12). Occupation status was classified into four groups: manual workers (including farmers, factory workers, and service workers), mental workers (including teachers, researchers, office assistants, and others technical staff), retirees and other kinds of workers (including students and unemployed persons).

Body mass index (BMI) was calculated as weight in kilograms divided by height in metres squared; BMI was classified into four groups according to the Chinese BMI classification criteria: underweight (BMI <18.5 kg/m^2^), normal (18.5≤ BMI <24 kg/m^2^), overweight (24≤ BMI <28 kg/m^2^), and obese (BMI ≥28 kg/m^2^)^52^. Smoking status included three groups: Never, Current, and Past. Never smokers were individuals who reported never having smoked up to 100 cigarettes. Current smokers were individuals who consumed any kind of tobacco products per day in the past 30 days. Past smokers were individuals who had smoked before but had quit at least three months prior^53^. Participants who consumed any kind of alcoholic beverage during the past year before the interview were defined as current drinkers. Physical exercise (excluding regular activity associated with daily life) status was divided into three levels: the ‘Never or rare’ group included individuals who never or seldom exercised; the ‘Exercise sometimes’ group included individuals who exercised one or two times per week; the ‘Often exercise’ group included individuals who exercised more than three times per week^54^.

### Ethics

The Health Bureau of Jilin province approved this project. The School of Public Health, Jilin University, organized the investigation. Each participants in the research signed written informed consent.

### Statistical analysis

The analysis of this study was conducted using SPSS version 22.0 statistical software (IBM Corp, Armonk, NY, USA). We used a weighted approach throughout the data analysis. An all-data weighted statistical analysis was implemented using the complex sample function in SPSS. The data was weighted by age, sex, and region (urban or rural) according to the demographic data of the statistical bureau of Jilin province to make more it representative of the 18~59 years old hypertensive patients in that province. The characteristics of participants were described by frequency and percentage. Descriptive analyses for non-normally distributed of quantitative data were described by median and interquartile range. The standardized rates of awareness, treatment and control of hypertension were weighted by age, sex, and region (urban or rural) in Jilin Province of China. The comparison of the prevalence of awareness, treatment and control among hypertensive patients in different categories were conducted by Rao-Scott Chi-square tests for complex samples. The regression analysis was first carried out by univariate regression analysis first. Factors that had a significant *P* value in the univariate regression were brought into the multivariable regression analysis. The multivariable logistic regression analysis was mandatorily adjusted for age, gender and region (urban or rural). Binary logistic regression analyses (Method: Forward: LR) were performed to identify potential independent factors associated with the awareness, treatment and control of hypertension. The significance threshold was 0.05.
